# Poly(*N*-Isopropylacrylamide)-Functional Silicon Nanocrystals for Thermosensitive Fluorescence Cellar Imaging

**DOI:** 10.3390/polym12112565

**Published:** 2020-11-01

**Authors:** Yiting Li, Lihui Zhang, Youhong Shi, Jialing Huang, Yaqiong Yang, Dengming Ming

**Affiliations:** 1 College of Food and Pharmaceutical Engineering, Nanjing Normal University, Nanjing 210023, China; liyiting01@163.com (Y.L.); zhanglihui@njnu.edu.cn (L.Z.); 2 College of Biological and Pharmaceutical Engineering, Nanjing Tech University, Nanjing 211816, China; 3 College of Pharmacy, Nanjing Tech University, Nanjing 211816, China; syh135846@126.com (Y.S.); 15195809957@163.com (J.H.)

**Keywords:** silicon nanocrystals, PNIPAAm, fluorescent imaging, thermoresponsive

## Abstract

Silicon nanocrystals (Si NCs) have received surging interest as a type of quantum dot (QD) due to the availability of silicon in nature, tunable fluorescence emission properties and excellent biocompatibility. More importantly, compared with many group II–VI and III–V based QDs, they have low toxicity. Here, thermoresponsive poly(*N*-isopropylacrylamide) (PNIPAAm)-functional Si NCs were firstly prepared for thermoresponsive detection of cancer cells. Si NCs were prepared under normal pressure with excellent water solubility. Then folic acid was bonded to the silicon nanocrystals through the reaction of amino and carboxyl groups for specific recognition of cancer cells. The folic-acid-modified silicon crystals (Si NCs-FA) could be modified by a one-pot copolymerization process into PNIPAAm nanospheres during the monomer polymerization process (i.e., Si NCs-FA-PNIPAAm) just by controlling the temperature below the lower critical solution temperature (LCST) and above the LCST. The results showed that the Si-FA-PNIAAm nanospheres exhibited not only reversible temperature-responsive on-off fluorescence properties, but also can be used as temperature indicators in cancer cells.

## 1. Introduction

Silicon nanocrystals (Si NCs) are emerging as a fascinating type of quantum dots for their unique properties, such as favorable biocompatibility, low toxicity, tunable fluorescence emission properties and good chemical stability [1,2]. Thus, they have shown promising potential in a number of fields, including fluorescent sensing [3], biological imaging [4], dynamic therapy [5], optoelectronic devices [6] and solar cells [7,8]. Intracellular temperature was closely related to cellular events [9]. Particularly, temperature variation can contribute to the intricate neurotransmitter release [10]. Moreover, cancer cell death by hyperthermia could potentially activate the immune system to fight against tumors [11]. Therefore, detection of intracellular temperature poses an important application in biomedicine.

Hybrids of functional polymers are fascinating for their tunable properties to obtain the unique characteristics of the components, including hybrid features ascribed to the cooperative effect between the polymer and Si NCs. Various methods for integrating Si NCs and polymers have been established [12,13,14,15]. Sacarescu et al. prepared a stable colloidal silicon nanocrystals-polysilane nanocomposite using microwave-activated coupling of organochlorosilanes with better stability for fluorescence detection [16]. Drawing on study correspondence to complex Si NCs, Hessel et al. encapsulated alkyl-terminated Si NCs into poly(maleic anhydride) hybrids, obtaining a water dispersible composite of a suitable size to get through the renal system and for biological imaging applications [17]. Samantha et al. used silicon quantum dot/poly(methyl methacrylate) in order to limit scattering and produce functional luminescent solar concentrators [18]. In addition, developing stimuli-responsive nanoparticles using poly(*N*-isopropylacrylamide) (PNIPAAm) have received promising interest [19,20,21]. The optical properties can be switched by external triggers, such as change of temperature, which the bare nanoparticles cannot achieve and are potential components for smart materials. The lower critical solutions temperature (LCST) of PNIPAAm is around 32 °C. Below the LCST, the intermolecular hydrogen bond in PNIPAAm chains is hydrated (swollen state). Above the LCST, the intramolecular hydrogen bond in PNIPAAm chains reversibly become hydrophobic (shrunken state). To our knowledge, this is the first time that thermoresponsive PNIPAAm-functional Si NCs were prepared for thermoresponsive detection of cancer cells.

Herein, Si NCs were prepared under normal pressure with excellent water solubility. Then folic acid was bonded to the silicon nanocrystals through the reaction of amino and carboxyl groups for specific recognition of cancer cells. Then the folic-acid modified silicon crystals (Si NCs-FA) could be modified by a one-pot copolymerization process into PNIPAAm nanospheres during the monomer polymerization process (denoted as Si NCs-FA-PNIPAAm) by simply controlling the preparation temperature below the LCST and above the LCST (at two different polymerization stages). The Si NCs-FA-PNIPAAm can be used in temperature sensing of cancer cells.

## 2. Experimental Section

### 2.1. Materials

3-Aminopropyltriethoxysllane (APTES, 98%, TCI Chemicals Co. Ltd., Shanghai, China), trisodium citrate dihydrate (AR, Sinopharm Chemical Reagent Co. Ltd., Shanghai, China), glycerol (AR, Shanghai Lingfeng Chemical, Shanghai, China), *N*-ethyl-*N’*-(3-dimethylaminopropyl) carbodiimide hydrochloride (EDAC, 99%, Shanghai Yuanye Biotechnology Chemical Co. Ltd., Shanghai, China), *N*-hydroxysuccinimide (NHS, 98%, Aladdin Chemicals, Shanghai, China), *N,N’*-methylenebis(acrylamide) (MBA, 99%, Aladdin Chemicals, Shanghai, China), *N*-isopropylacrylamide (NIPAAm, 98%, Aladdin Chemicals, Shanghai, China), folic acid (FA, 97%, Huixing biochemical reagent Co. Ltd., Shanghai, China), potassium peroxydisulfate (KPS, AR, Xilong Scientific, Shantou, China), 3-(4,5)-Dimethylthiahiazo(-2-yl)-3,5-diphenytetrazoliumromide (MTT, BDH laboratory Supplied, Poole, Dorset, England). All reagents were used when received without further purification.

### 2.2. Synthesis of PNIPAAm-Functional Si NCs

#### 2.2.1. Synthesis of Si NCs

Trisodium citrate dihydrate and APTES were chosen as reduction reagent and silicon source, respectively. Glycerol was utilized as solvent, and water-dispersible silicon quantum dots were synthesized according to the previous method [22]. The details were as follows: sodium citrate (1.229 mmol) was added to a three-necked round-bottomed flask containing glycerol (8 mL) under the protection of argon after vigorous stirring for 20 min at room temperature and normal pressure. Then APTES (2 mL) was added for continually stirring for 10 min at 185 °C for 1.5 h. When the solution changes from colorless to yellow and has bright blue fluorescence under the ultraviolet lamp, indicated the formation of Si NCs. Dialysis bags (MWCO:3500 Da) were used to remove the remaining APTES and sodium citrate. Pure Si NCs were obtained by freeze-drying.

#### 2.2.2. Synthesis of Folic Acid Modified Silicon NCs (Si NCs-FA)

The Si NCs modified with folic acid were synthesized according to the previously reported method with some modification [23]. Here, FA was covalent reacted with the amino-group in the Si NCs. The procedure for preparing folate modified silicon Si NCs were as follows: EDAC (16 mg) and NHS (10 mg) were dissolved in purified water (4 mL), Si NCs (5 mg) were added to the solution, stirring at 30 min under nitrogen protection. Then FA (0.5 mg/mL, 2 mL) was added to the solution. After mixing for 3 h, the crude folic acid modified Si NCs were obtained. Dialysis bags (MWCO: 8000–14,000 Da) were used to remove the remaining reactants.

#### 2.2.3. Synthesis of Temperature Sensitive *N*-Isopropylacrylamide (PNIPAAm)-Coated Si NCs-FA (Si NCs-FA-PNIPAAm)

Temperature-sensitive Si NCs-FA-PNIPAAm was prepared by free radical copolymerization. The details were as follows: NIPAAm (310 mg) and MBA (3.4 mg) were added to a three-necked round-bottomed flask containing purified water (20 mL) under the protection of argon and stirred for 30 min. Then Si NCs-FA (30 mL) and KPS (0.1294 mM, 2 mL in purified water) were added to the above solution, and continue stirring at another 30 min under room temperature. Next, the temperature of solution was raised to 70 °C for 4 h. The tiny argon bubbles should be kept uniform and continuous in the whole process. The pure Si NCs-FA-PNIPAM was obtained after three repetitions of centrifugation at 12,000 rpm for 20 min when the solution was cooled to room temperature. At last, the precipitates were redispersed in pure water.

### 2.3. Characterization

**Scheme 1:**
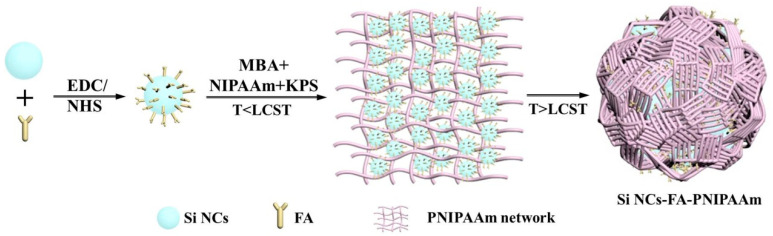
Preparation of thermoresponsive Si NCs-FA-PNIPAAm nanoparticles.

A fluorescent spectrometer (F-7000, Hitachi, Japan), a FT-IR spectrometer (Thermo, Nicolet iS50, USA), a transmission electron microscope (TEM) (Jeol Jem-2100F, Japan) and a UV-vis spectrometer (UH-5300, Hitachi, Japan) were used. Dynamic laser scattering (DLS) spectrometer (Malvern Nano ZSE Instrument) was chosen to characterize the particle sizes of nanoparticles. A confocal laser scanning microscopy (CLSM, Zeiss LSM 710, Germany) with excitation at 400 nm was selected to conduct the cell imaging. A Rigaku D/Max-3C X-ray diffractometer with an Inel CPS 120 hemispherical detector was employed for measuring the X-ray diffraction (XRD) pattern using CuKα1 radiation (λ = 1.54 Å) with the operating conditions at 40 kV and 30 mA. 3D-MAX was also used to depict the Scheme 1.

### 2.4. Cell Culture

Hela cells and MCF-7 cells were purchased from Jiangsu keyGEN BioTECH (Nanjing, China). The cells were cultured according to the previous method [24].

### 2.5. Cell Biocompatibility Assay

The 3-(4,5)-dimethylthiahiazo(-2-yl)-3,5-diphenytetrazoliumromide (MTT) assay was selected to evaluate the biocompatibility of Hela and MCF-7 cells. The experiment process was according to the previous method [24]. (A_test_/A_control_) × 100% was used to calculate cell viability (%).

### 2.6. Cell Imaging

Hela cells and MCF-7 were seeded in a six-well plate with cover glass and incubated for 24 h, respectively. The cells were treated with 200 μg/mL of the Si NCs-FA-PNIPAAm for 1 h after being washed three times with PBS.

## 3. Results and Discussion

### 3.1. Characterization of Fluorescent Probes

Temperature-sensitive fluorescent probes (Si NCs-FA-PNIPAAm nanoparticles) were synthesized by free radical copolymerization. Si NCs, folic acid and NIPAAm were employed as the fluorescent signal, cancer target and temperature sensing medium, respectively. Si NCs were synthesized using APTES as silicon source under normal pressure with glycerol as solvent. Then folic acid was bonded to Si NCs through the reaction of amino and carboxyl groups for specific recognition of cancer cells. The folic-acid modified silicon crystals (Si NCs-FA) could be modified by a one-pot copolymerization process into PNIPAAm nanospheres during the monomer polymerization process (i.e., Si NCs-FA-PNIPAAm) by controlling the preparation temperature below LCST and above the LCST (at two different polymerization stages). In the first stage, KPS was chosen to initiate NIPAAm monomers below the LCST (25 °C) to form the PNIPAAm networks with Si NCs-FA. The network and Si NCs-FA were more homogeneous, and Si NCs-FA particles were uniformly dispersed throughout [25]. In the second stage, the spheres were obtained after the temperature was raised above the LCST (70 °C), the PNIPAAm networks undergo an abrupt transition with the chains collapsing. The network became heterogeneous, and polymer-rich and polymer-poor phases were created. In this process, hydrophobic forces, hydrogen bonding and self-assembly may contribute to the Si NCs-FA becoming close-packed in the spheres [26,27]. Therefore, the Si NCs-FA nanoparticles were entrapped in the PNIPAAm network (Scheme 1).

**Figure 1:**
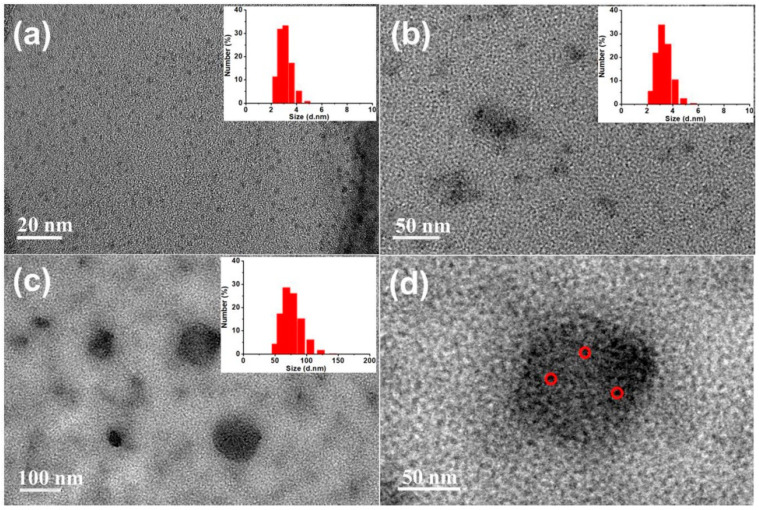
TEM images of Si NCs (**a**), Si NCs-FA (**b**), Si NCs-FA-PNIPAAm (**c**) and enlarged Si NCs-FA-PNIAAm in Figure 1c (**d**).

The diameters of Si NCs, Si NCs-FA and Si NCs-FA-PNIPAAm were 3.1, 3.3 and 75.3 nm, respectively which were detected by DLS (the inset of Figure 1). As shown in the Figure 1, the Si NCs exhibited good monodispersity with spherical particles (Figure 1a). Meanwhile, the high-resolution TEM image of Si NCs was also measured (Figure S1 in supplementary materials), which showed that Si NCs showed a well-resolved lattice spacing of 0.31 nm, which is also consistent with the reported literature [28]. Combined with the observation of characteristic peaks (111) for Si NCs in their XRD patterns (Figure S2 in supplementary materials), the results indicated the successfully preparation of Si NCs. The diameter of Si NCs-FA-PNIPAAm networks was from 33 to 108 nm detected by TEM (Figure 1c), and, as shown in the enlarged TEM image of Si NCs-FA-PNIAAm (Figure 1d), Si NCs-FA was entrapped into the PNIPAAm network. The photos Si NCs, Si NCs-FA and Si NCs-FA-PNIPAAm were also showed in the Figure S3 (in supplementary materials). The results showed that they were yellow powder.

**Figure 2:**
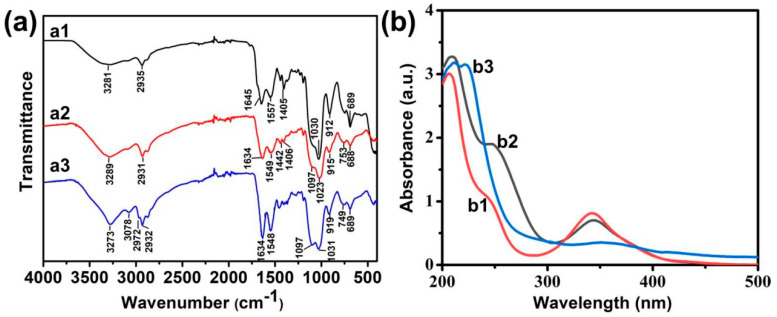
(**a**) FT-IR spectroscopy of Si NCs (a1), Si NCs-FA (a2), Si NCs-FA-PNIPAAm (a3); (**b**) UV-vis spectra of Si NCs (b1), Si NCs-FA (b2), Si NCs-FA--PNIPAAm (b3), the concentration of them was 5 μg/mL.

The surface structure of the prepared Si NCs was further confirmed by FT-IR spectroscopy (Figure 2a1). The absorbance peak at 1030 cm^−1^ was assigned to the vibrational stretching of Si−O bond. The bending vibration and stretching vibration of the N−H bond belonging to the absorption peaks were at 1645 and 3281 cm^−1^. These results show that the synthesized Si NCs were rich in amino groups. Therefore, covalent combination of carboxyl and amino groups can modify folic acid onto the surface of Si NCs. As shown in FT-IR spectra, the characteristic amide bands were around 1634 cm^−1^ (amide I) and 1549 cm^−1^ (amide Ⅱ) of Si NCs-FA (Figure 2a2) and they were stronger than Si NCs (Figure 2a1), which confirmed that the successful incorporation of folic acid. Moreover, the successful coating of NIPAAm was confirmed by the stronger absorption of amide bonds (3273 cm^−1^) and the appearance peak at 2972 cm^−1^ attributed to the –CH_3_ in Si NCs-FA-PNIPAAm (Figure 2a3).

The existence of folic acid on Si NCs-FA and Si NCs-FA-PNIPAAm was also confirmed by the presence of the characteristic strong peak of folic acid (around 280 nm) compared with the Si NCs in their UV-vis spectra (Figure 2b1,b2). In addition, the broadening of the absorption peak at 360 nm in UV-Vis spectra (Figure 2b3) was attributed to the successful coating of Si NCs in Si NCs-FA-PNIPAAm.

### 3.2. Optical Properties of the Fluorescent Probes

**Figure 3:**
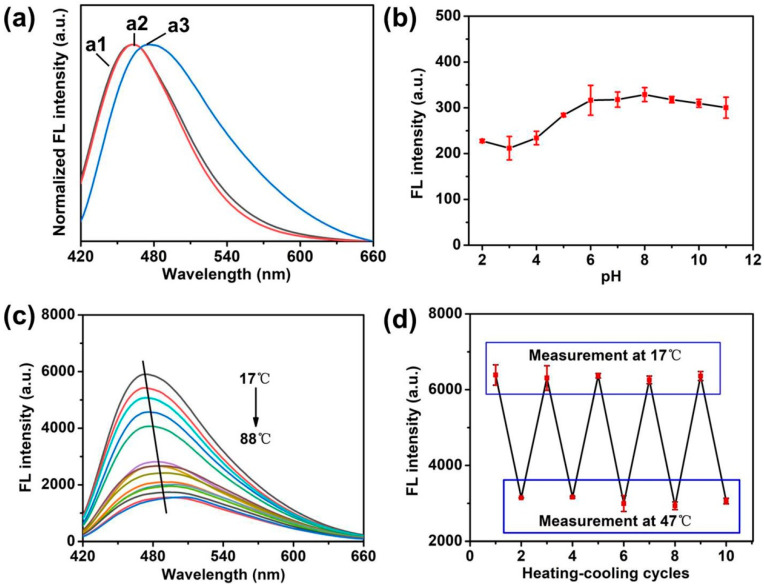
(**a**) The emission spectra of Si NCs (a1), Si NCs-FA (a2) and Si NCs-FA-PNIPAAm (a3). (**b**) Dependence of the fluorescence intensity of Si NCs-PNIAAm on surrounding pH values. Concentration: 0.2 mg/mL of Si NCs-FA-PNIPAAm was dissolved in 0.01 mol/L phosphate solution at different pH; excitation wavelength: 404 nm; emission wavelength: 476 nm. (**c**) Effects of temperature on the fluorescence emission spectra of the Si NCs-FA-PNIPAAm (10 mg/mL in purified water); excitation wavelength: 404 nm. (**d**) Fluorescence intensity measured during repeated heating (47 °C) and cooling (17 °C) cycles of the Si NCs-PNIAAm.

The optical properties of Si NCs, Si NCs-FA and Si NCs-FA-PNIPAAm were studied and compared. Figure 3a shows the normalized fluorescence emission spectra of Si NCs, Si NCs-FA and Si NCs-FA-PNIPAAm. The strong fluorescence at 462 nm in the fluorescence emission spectrum indicated that the Si NCs was successfully synthesized. Compared with the Si NCs, the maximum emission of Si NCs-FA had a little shift. The maximum emission of Si NCs-PNIPAAm showed a little redshift of 14 nm, which may be caused by increased polarity in Si NCs-PNIPAAm and the increase of particle size caused by their modification. In addition, the Si NCs-PNIPAAm showed super fluorescence around physiological pH (Figure 3b). On the other hand, Si NCs and Si NCs-FA were also studied with the increasing of pH (Figures S4 and S5 in supplementary materials). The results indicated that the Fluorescence intensity (FL) of both Si NCs and Si NCs-FA was changed with the different pH, which is useless for their pH-sensitive application. Furthermore, the fluorescence emission spectra of Si NCs-PNIPAAm in the temperature range from 17 to 88 °C showed thermosensitive properties (Figure 3c). The results showed that the FL showed a sharp decrease from 17 to 42 °C, but the FL showed a slow decrease from 42 to 88 °C. However, the FL intensity of Si NCs and Si NCs-FA keep almost constant with the increasing of temperature (Figures S6 and S7 in supplementary materials). The small change of FL intensity of Si NCs-FA was caused by the FA, which have a small effect on FL depending on temperature [29,30]. Meanwhile, the FL intensity of FA was very low compared with the FL of Si NCs and Si NCs-FA (Figure S8 in supplementary materials), and the FL intensity of FA (1 mg/mL aqueous solution) was about 40 times lower than the FL of Si NCs-FA (1 mg/mL aqueous solution), which can rule out the possibility that the observed fluorescence change of Si NCs-FA might arise from folic acid. These results also demonstrated the changing of FL was mostly caused by PNIPAAm. Meanwhile, the emission peaks of Si NCs-FA-PNIPAAm shift to longer wavelengths with the increasing of the temperature. These results may be caused by breaking of hydrogen bonds between the monomer side groups and water molecules, and so PNIPAAm undergoes changes in conformation [31]. As the result, the microenvironment, especially the directric constant of nanocrystals embedded in the PNIPAAm network changes [25]. As observed in Figure 3d, the Si NCs-FA-PNIPAAm showed obvious temperature dependence and the fluorescence intensity almost restored to the original value after five heating and cooling cycles, indicating that there is a connection between the fluorescence property and the volume transformation of the network. The repeated hot and cold cycles showed that Si NCs in the PNIPAAm gels had little influence on the polymer characteristics of the temperature-induced phase transition.

### 3.3. Application of the Fluorescent Probes

**Figure 4:**
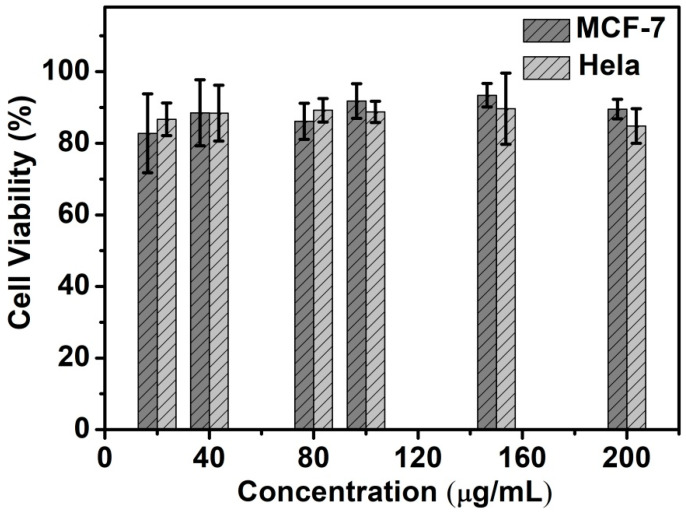
In vitro biocompatibility of Hela and MCF-7 cells after co-cultured with the Si NCs-FA-PNIPAAm for 24 h.

The biocompatibility of Si NCs-FA-PNIPAAm was evaluated through counting viability and observing morphology of the Hela cells and MCF-7 cells. MTT assay was used to quantify the cytotoxicity of Si NCs-PNIAAm nanoparticles. The cellular viability of Hela cells and MCF-7 retained about 85%, indicating that Si NCs-FA-PNIPAAm had good compatibility within the concentration range of 20–200 μg/mL (Figure 4). The Hela cells and MCF-7 cells also remained good adherence with strong refraction and fusiform, which indicated that cells were in good condition.

**Figure 5:**
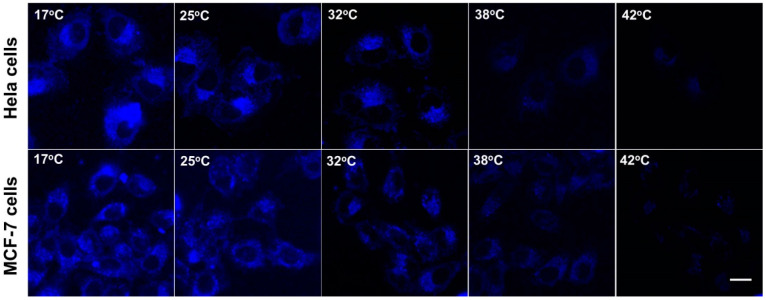
Confocal fluorescence imaging of Hela cells and MCF-7 cells after being stained with Si NCs-FA-PNIPAAm (the scale bar is 20 μm).

To explore the application of the Si-NCs-PNIPAAm for thermosensitive detection of cancer cells, Hela cells and MCF-7 cell were chosen as the recognition model. The HeLa cells and MCF-7 were used to conduct the fluorescence imaging of intracellular temperature variation. Overexpression of folic acid receptors on the tumor surface endows folic acid with the ability to recognize tumor cells [32,33]. As shown in Figure 5, the representative fluorescence images with temperature increasing from 17 to 42 °C were shown in Figure 5. The images revealed that there was characteristic temperature-dependent fluorescence intensity from the cells in the range of 17 to 42 °C. Meanwhile, the semiquantitative analysis of mean fluorescence intensity of as-prepared Si NCs-FA-PNIPAAm particles was shown in Figure S9 (in supplementary materials). It is demonstrated that the mean intensity decreased with increasing temperature. These results also confirmed that the fluorescence intensity of Si NCs-PNIPAM particles becomes lower, which indicated their potential application on thermosensitive detection of cancer cells.

## 4. Conclusions

We firstly designed the PNIPAAm-functional Si NCs for thermoresponsive detection of cancer cells. The polymeric matrix was prepared by controlling the reaction temperature at two different polymerization stages. The results showed that the Si NCs-FA-PNIAAm nanospheres exhibited not only reversible temperature-dependent on-off fluorescence properties, but also can be used as temperature indicators in cancer cells.

## Supplementary Materials

The following are available online at https://www.mdpi.com/2073-4360/12/11/2565/s1, Figure S1: High-resolution TEM image of the Si NCs; Figure S2: X-ray powder diffraction pattern of the Si NCs-PNIPAAm; Figure S3: Photos of Si NCs, Si NCs-FA and Si NCs-FA-PNIPAAm in solid; Figure S4: Dependence of the fluorescence intensity of Si NCs on surrounding pH values; Figure S5: Dependence of the fluorescence intensity of Si NCs-FA on surrounding pH values; Figure S6: Effects of temperature on the fluorescence emission spectra of the Si NCs; Figure S7: Effects of temperature on the fluorescence emission spectra of the Si NCs-FA; Figure S8: The emission spectra of Si NCs (a) (1 mg/mL in pure water), Si NCs-FA (1 mg/mL in pure water) (b) and FA (1 mg/mL) (c); Figure S9: Normalized mean fluorescence intensity of Si NCs-FA-PNIPAAm at Hela and MCF-7 cells corresponding to the confocal fluorescence microscope in Figure 5.
