# Supplementary material for: Poly(N-Isopropylacrylamide)-Functional Silicon Nanocrystals for Thermosensitive Fluorescence Cellar Imaging
Source: Polymers (Basel). 2020 Nov 1;12(11):2565. doi: 10.3390/polym12112565 (PMC7693885; doi:10.3390/polym12112565)
Supplement: Supplementary file 1 [file polymers-12-02565-s001.pdf]

Supporting Information

Poly(*N*-Isopropylacrylamide)-Functional Silicon Nanocrystals for Thermosensitive Fluorescence Cellar Imaging

Yiting Li [1,2,#], Lihui Zhang [1,#], Youhong Shi [3], Jialing Huang [3], Yaqiong Yang [2,*] and Dengming Ming [1,*]

[1] College of Food and Pharmaceutical Engineering, Nanjing Normal University, Nanjing 210023, PR China
[2] College of Biological and Pharmaceutical Engineering, Nanjing Tech University, Nanjing 211816, PR China
[3] College of Pharmacy, Nanjing Tech University, Nanjing 211816, PR China
# Yiting Li and Lihui Zhang contributed equally.
* Correspondence: yangyq@njtech.edu.cn (Y.Y.); dming@njtech.edu.cn (D.M.)

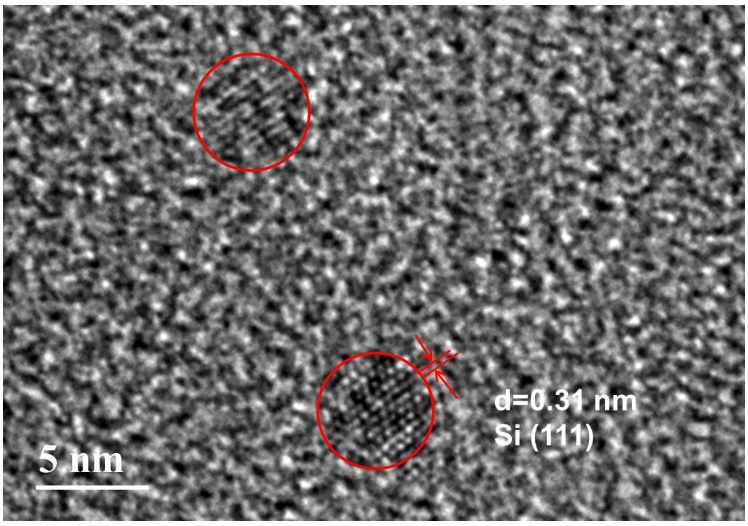

Figure S1 High-resolution TEM image of the Si NCs.

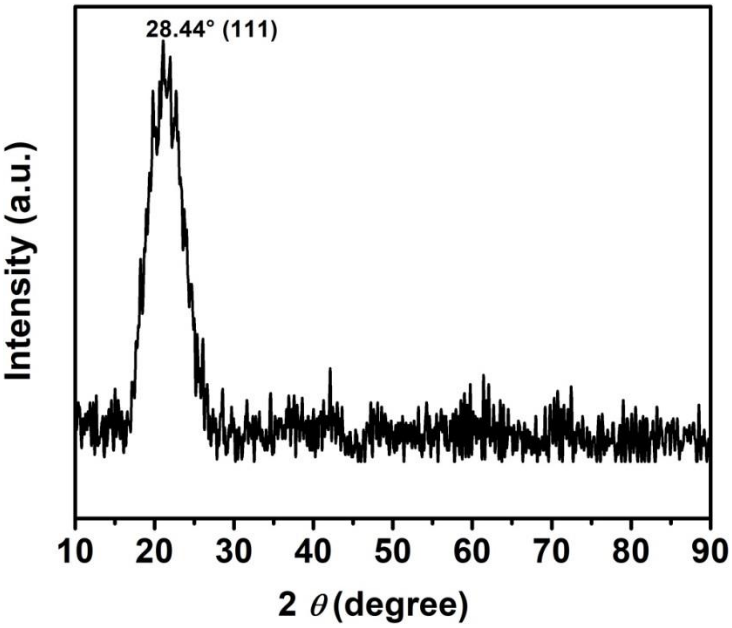

Figure S2 X-ray powder diffraction pattern of the Si NCs-PNIPAAm.

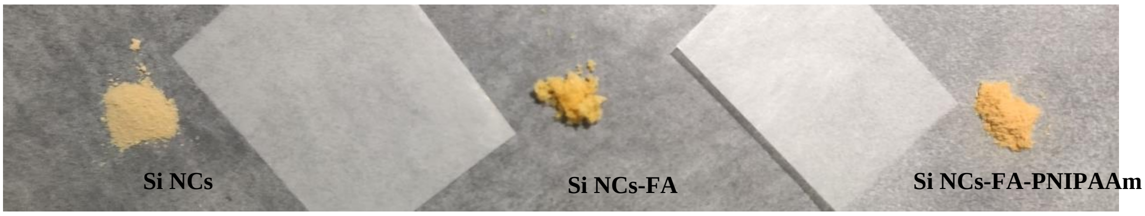

Figure S3 Photos of Si NCs, Si NCs-FA and Si NCs-FA-PNIPAAm in solid.

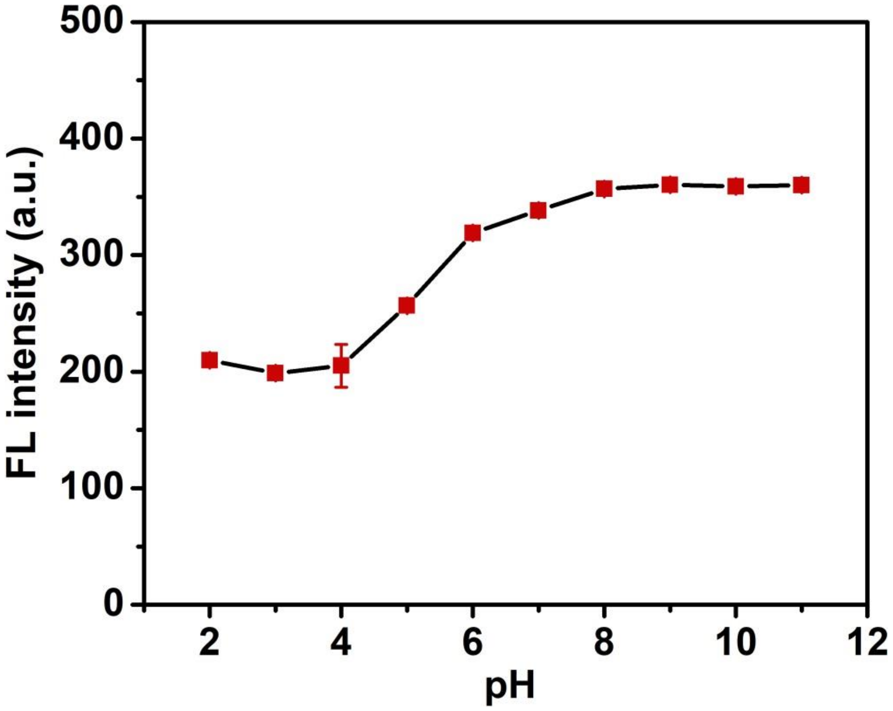

Figure S4 Dependence of the fluorescence intensity of Si NCs on surrounding pH values.

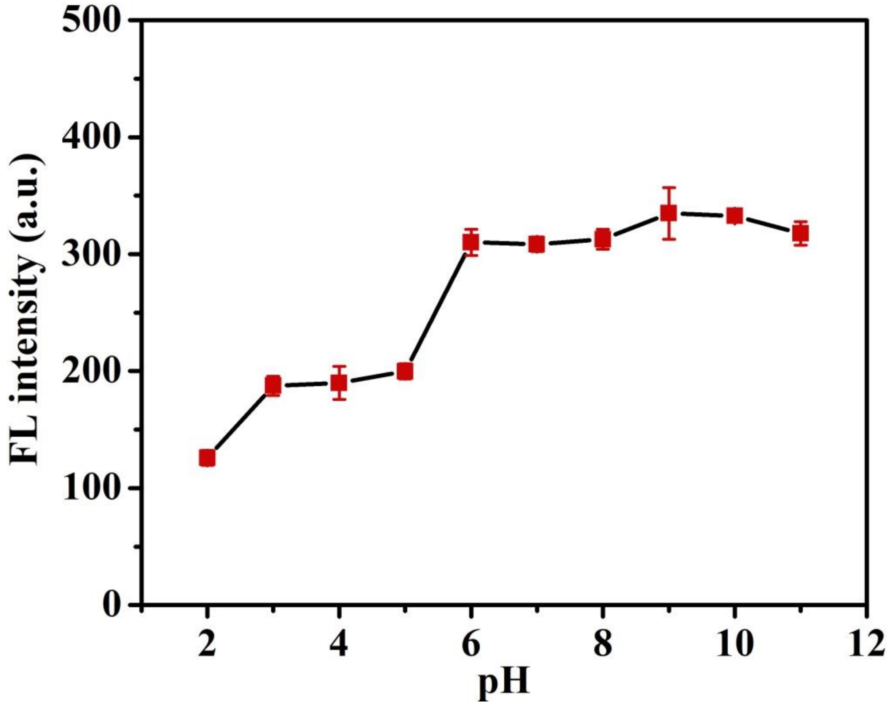

Figure S5 Dependence of the fluorescence intensity of Si NCs-FA on surrounding pH values.

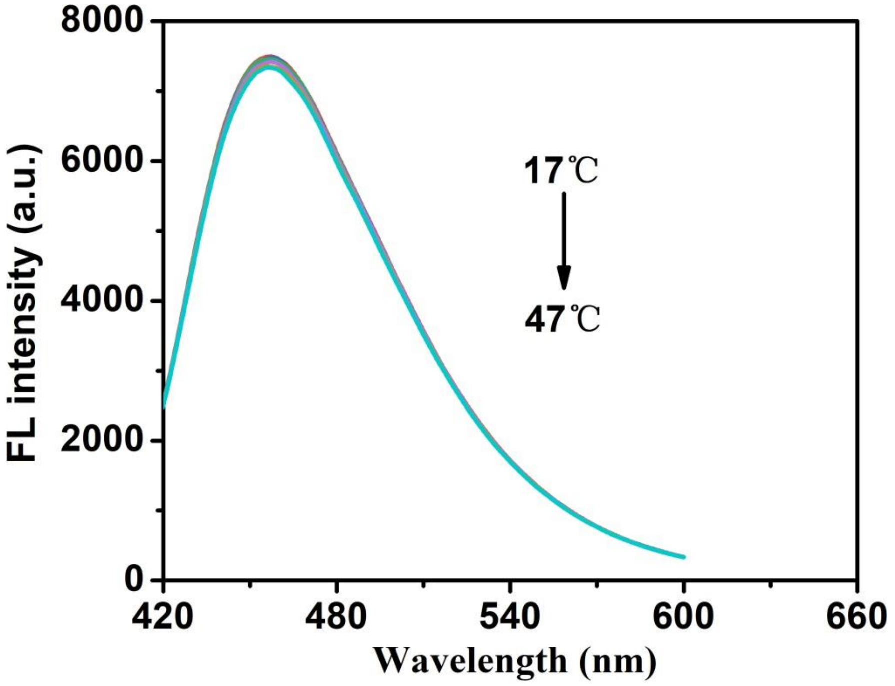

Figure S6 Effects of temperature on the fluorescence emission spectra of the Si NCs.

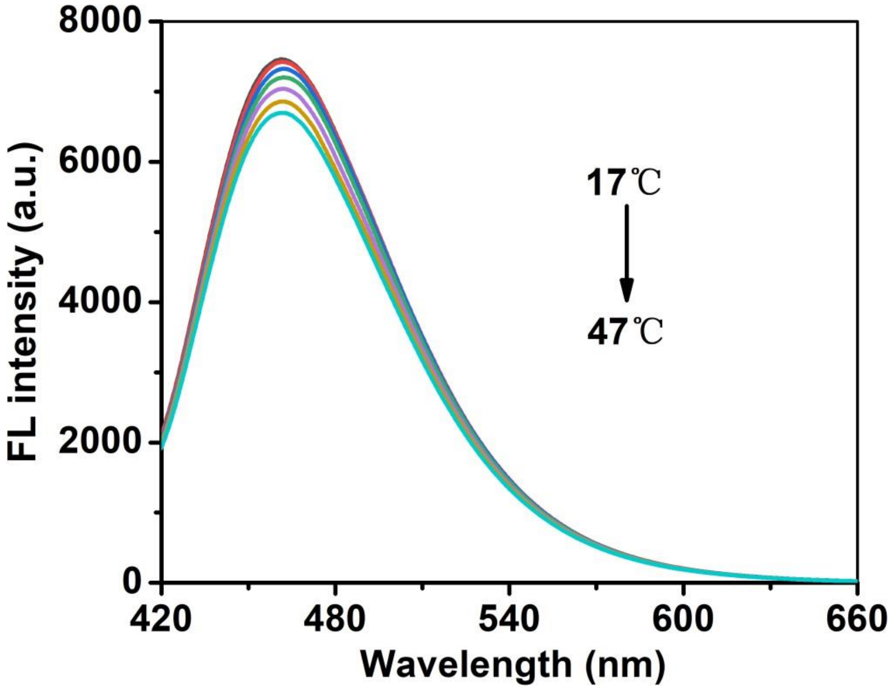

Figure S7 Effects of temperature on the fluorescence emission spectra of the Si NCs-FA.

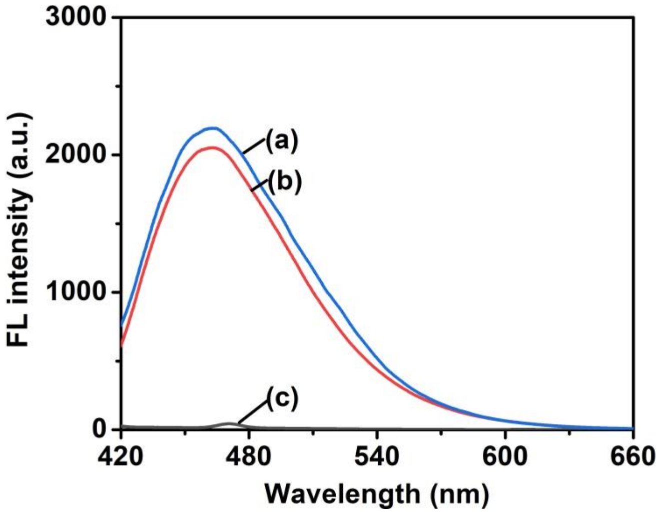

Figure S8 The emission spectra of Si NCs (a) (1 mg/mL in pure water), Si NCs-FA (1 mg/mL in pure water) (b) and FA (1 mg/mL) (c).

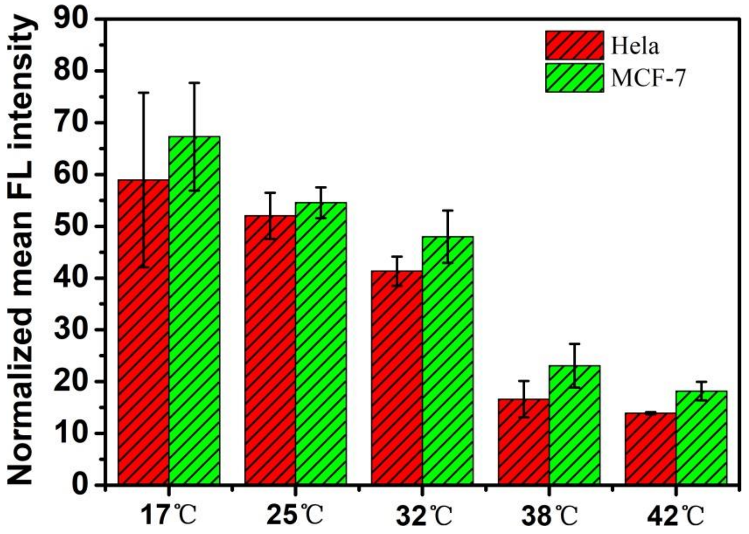

Figure S9 Normalized mean fluorescence intensity of Si NCs-FA-PNIPAAm at Hela and MCF-7 cells corresponding to the confocal fluorescence microscope in Figure 5.
